# Editorial: Current trends and approaches in the comprehensive evaluation of coronary artery disease

**DOI:** 10.3389/fcvm.2023.1306413

**Published:** 2023-10-23

**Authors:** Gianluca Rigatelli, Dobrin Vassilev, Robert Gil

**Affiliations:** ^1^ UOS Emodinamica e Cardiologia Interventistica, Ospedali Riuniti Padova Sud, AULSS6 Euganea, Monselice, Italy; ^2^ Department of Cardiology, University of Ruse, Ruse, Bulgaria; ^3^ Invasive Department, Centre of Postgraduate Medical Education, Warsaw, Poland

**Keywords:** coronary artery disease, atherosclerosis, diagnostic, therapy, pathophysiology

**Editorial on the Research Topic** Current trends and approaches in the comprehensive evaluation of coronary artery disease

Coronary artery disease (CAD) continues to be a significant cause of mortality worldwide in the field of chronic degenerative diseases. For this reason, any attempt aimed to understanding its pathophysiologic basis, proper management, and therapy is worthy of efforts in order to improve both diagnosis and treatment outcomes. The present focused issue of Frontiers in Cardiovascular Medicine aims to provide an insight on the current evaluation of CAD by compiling the latest and most up-to-date research studies. The included papers can be broadly categorized as follows: (a) studies pertaining to molecules and biomarkers associated with CAD that are useful for an early diagnosis and understanding of its pathophysiology; (b) studies examining the prognostic factors capable of predicting clinical outcomes; and (c) practical clinical studies focusing on treatment methods such as drugs and percutaneous interventions.

Among the initial set of papers, Sun et al. explored the relationships between plasma Vitamin B5 and CAD, suggesting that plasma vitamin B5 has an L-shaped relationship with CAD, with a threshold at approximately 40.95 ng/ml. Intriguingly, the observed association was influenced by smoking. Zhang et al. reviewed the role of suppression of tumorigenicity 2 (ST2), a member of the interleukin 1 (IL-1) receptor family, and formally known as interleukin 1 receptor-like 1 (IL1RL-1), as a potential biomarker and prognostic factor in the diagnosis and management of CAD. This biomarker is indicative of the extent of plaque accumulation and has the potential to predict the occurrence of no-reflow events, as well as the prognosis of patients. Bil et al. investigated the role of distribution width (DW) and red cell distribution width (RDW) in the diagnosis of coronary microvascular spasm in patients undergoing acetylcholine test. The findings of the study indicated a correlation between both DW and RDW and poor prognosis in patients over a 5-year period.

In an elegant meta-analysis, Zhang et al. showed how an elevated blood CXCL12 level was associated with an increased occurrence of MACEs in patients with CAD, and could potentially serve as an important prognostic index for CAD. Similarly, Yan et al. demonstrated that high white blood cell (WBC) count was associated with the risk of occurrence of all-cause mortality and cardiac mortality, myocardial infarction, stroke, unplanned revascularization, and major adverse cardiovascular and cerebrovascular events following PCI. On the contrary, Liu et al. utilized an angiography-derived index of microcirculatory resistance to demonstrate that elevated levels of syndecan-1, a component of endothelial glycocalyx (EG) that plays a crucial role in maintaining microvascular homeostasis, are independently associated with the presence of coronary microvascular dysfunction and an impaired microvascular vasodilatory capacity in patients with suspected CAD.

In the second group of papers, Yoshioka et al. explored the prognostic impact of incident left ventricular systolic dysfunction following myocardial infarction, suggesting how incident LV systolic dysfunction during the chronic phase following acute myocardial infarction (AMI) was significantly associated with long-term adverse outcomes. Similarly, Tang et al. showed that LAFI was a strong and independent predictor of adverse events and can be used for risk stratification in patients with AMI treated with PCI. In a retrospective study conducted by Fang et al., it was suggested that urea nitrogen, Killip class II–IV, LVEF, and NT-ProBNP are independent factors associated with in-hospital MACE after PCI in STEMI patients, and nomogram models constructed based on the aforementioned factors have high predictive efficacy and feasibility.

The third and last group of papers included more clinically oriented studies. Sheiban et al. provided an insight of the treatment of coronary bifurcation using either one or two stent strategy. The rescue salvaging of the side branch in their patients was found to be associated with a higher rate of 3-year target lesion failure (TLF), particularly when predilated. Han et al. reported the results of the China Registry on NSTEMi patients, suggesting that the early invasive strategy did not reduce the incidence of MACEs and mortality within 30 days compared with the delayed invasive strategy. Will et al. confirmed that the left transradial angiography is associated with a higher first-pass catheter success rate for coronary artery angiography compared with the right transradial approach. Legutko et al. suggested that in discrepant resting full-cycle ratio (RFR)/flow fractional reserve (FFR) vessels, coronary microvascular dysfunction is more prevalent than in concordant RFR/FFR measurements. According to a study of Li et al., the implementation of a risk stratification program utilizing the Barthel index during hospitalization has the potential to predict outcomes in acute coronary syndrome (ACS) patients.

In terms of CAD treatment, Yin et al. confirmed that enhanced external counter pulsation has poor compliance. Conversely, a meta-analysis conducted by Ma et al. has demonstrated that Colchicine has a positive effect in reducing the incidence of MACE, MI, stroke, and revascularization, but may increase the risk of gastrointestinal complications and diarrhea. Yu et al. have presented an interesting insight on the anomalous origin of the coronary artery from the pulmonary artery in patients who have undergone mitral valve surgery. In addition to the aforementioned studies, Wang et al. conducted a review of the available evidence pertaining to the use of artificial intelligence in developing diagnostic models for coronary artery disease with imaging markers.

The studies presented are intriguing and surely able to stimulate the interest of readers. The clinical insights provided in each publication are anticipated to be incorporated into the research and clinical practices of cardiovascular professionals around the world.

